# Ileal adenocarcinoma in a young pregnant woman: A rare case report

**DOI:** 10.3389/fonc.2023.1066153

**Published:** 2023-01-25

**Authors:** Chao Xiao, Qijun Cheng, Chengjian Cao, Xue Xiao, Yutao Zhang

**Affiliations:** ^1^ Department of Obstetrics and Gynecology, West China Second University Hospital, Sichuan University, Chengdu, China; ^2^ Department of Obstetrics and Gynecology, The First People's Hospital of Zigong, Zigong, China; ^3^ Clinical Research Laboratory & Department of Clinical Examination, The First People's Hospital of Zigong, Zigong, China; ^4^ Department of Pathology, The First People’s Hospital of Zigong, Zigong, China

**Keywords:** abdominal pain, case report, ileal neoplasms, intestinal obstruction, pregnancy, vomiting

## Abstract

Primary intestinal malignancies account for only 1%–3% of all malignant gastrointestinal tumors. Adenocarcinomas are uncommonly located in the ileum. Ileal adenocarcinoma (IA) is rare and difficult to diagnose because of its location. IA is common in older men and rare in young pregnant women. A 23-year-old pregnant woman was hospitalized several times for repeated vomiting and abdominal pain. Her symptoms were relieved after symptomatic treatment. She exhibited no typical manifestations of intestinal obstruction, such as abdominal distension, difficulty passing gas and defecation. Unfortunately, she was misdiagnosed with acute gastroenteritis. On the second day after delivery, the patient stopped passing gas and computed tomography (CT) revealed an intestinal obstruction. She was treated as paralytic ileus. However, in view of failed conservative management, she was decided for an exploratory laparotomy. A malignant ileal tumor 5cm from the ileocecal valve was found incidentally and was surgically excised accompanied with End-to-side anastomosis of ileal and transverse colon. The operation lasted 195 minutes. Pathological examination revealed an IA. Pregnant woman who experience symptoms of intestinal obstruction should be alert to the possibility of malignancy in the small intestine. IA is an insidious tumor in pregnant women. An “IA triad” can be defined as refractory vomiting, vague abdominal pain, and weight loss (or inadequate weight gain in pregnant women). Pregnant women with an IA triad should undergo investigation with endoscopy or, if necessary, magnetic resonance imaging (MRI).

## Introduction

Primary intestinal malignancies account for only 1%–3% of all malignant gastrointestinal tumors. By subtype, 30%–40% are adenocarcinomas, 35%–44% are neuroendocrine tumors, 10%–20% are lymphomas, and 12%–18% are gastrointestinal stromal tumors (1). Adenocarcinomas are usually located in the duodenum but rarely in the jejunum and ileum (2). The diagnosis of intestinal carcinoma is usually delayed by more than 6 months because the clinical manifestations are nonspecific (3). Ileal adenocarcinoma (IA) is rare and difficult to diagnose because of its covert location. Takahashi et al. (4) reported 17 cases of early-stage IA during the period 1996–2016. The mean age of these patients was 66.1 years, the youngest being 55 years, and 15 of them were male. Thus, IA is more common in older men. However, ileum adenocarcinoma is rare in young patients, especially in young pregnant woman. Here we report the first case of IA in a young pregnant woman.

## Case

A 23-year-old woman presented to our department at 33 and 3/7 gestational weeks (GWs) of her first pregnancy, complaining of vaginal bleeding for 2 days and vomiting and abdominal pain for 10 h.

She experienced nausea and severe vomiting (approximately 5–10 times a day) during the first trimester. The vomiting did not stop at the end of the first trimester but was less severe than before. Weight gain was approximately 5 kg since the beginning of her pregnancy. Routine screening during her pregnancy suggested that the patient was heterozygous for the alpha-thalassemia gene, and her hemoglobin value fluctuated between 85 and 105 g/L (normal value: 110 g/L). At 26 GWs, she was hospitalized because of vaginal bleeding and exacerbated vomiting. Her diagnosis was threatened premature labor and acute gastroenteritis. She was given symptomatic treatment (fluid rehydration and antiemetics). The vomiting abated and the vaginal bleeding stopped.

At 30 and 5/7 GWs, the patient was admitted to our department for abdominal pain and recurrent exacerbated vomiting. She was diagnosed with acute cholecystitis based on a gallbladder stone revealed by ultrasonography and abnormal neutrophil counting. Antibiotics and antiemetics were prescribed accordingly. Dexamethasone was also prescribed to promote fetal lung maturation. Her symptoms abated quickly. At 33 and 3/7 GWs (January 7, 2021), the patient experienced lower abdominal pain and vaginal bleeding after vomiting. The vomiting, abdominal pain, and vaginal bleeding became worse and she was again hospitalized.

Upon examination, the length of the gravid uterus was 26 cm, the abdominal circumference was 78 cm, and the cervix was dilated at 1 cm. The fetal heart rate was 130 beats per minute. No tenderness in the abdomen was obvious. Tests for polypeptides, antibodies against autoimmune hepatitis, and antibodies against hepatitis E, B, and C showed negative results. The amylase level was normal. The blood test results are listed in **Table 1**. Ultrasonography revealed a strong echogenic accumulation of 3.0 × 0.7 cm in the gallbladder cavity and a fetus whose size corresponded to the number of GW.

**Table 1:** Timeline of test results and the treatment process.

Test time	ALT (U/L)	AST (U/L)	TBA (umol/L)	TBIL (umol/L)	HGB (g/L)	WBC (10^9/L)	NEUT (%)	Treatments or Events
Jan. 9	152	128	1.7	9.7	105	5.66	85.3	Dexamethasone; Fluid infusion; Glutathione; Nifedipine
Jan. 12	247	157	2.3	9.1	99	4.77	71.2	Pethidine hydrochloride
Jan.14	224	142	1.9	8.7	97	3.97	60.9	Chinese traditional medicine
Jan. 17	243	161	2	10	NA	NA	NA	Ursodeoxycholic acid; Sucralfate suspension
Jan. 20	NA	NA	NA	NA	NA	NA	NA	Vaginal delivery of a healthy boy
Jan. 21	NA	NA	NA	NA	NA	NA	NA	Stops defecating
Jan. 22	229	162	10.3	8.8	NA	NA	NA	Polyene phosphatidyl choline; Exhaustion stops; Small intestine obstruction; Transferred
Jan. 25	NA	NA	NA	NA	NA	NA	NA	Laparotomy for unrelieved obstructive symptoms
Jan. 26	86	36	1.3	8.2	90	5.8	76.8	Parecoxib sodium; Glutathione
Feb. 2	19	19	0.7	8	85	4.01	56.1	Discharged without discomfort

Jan., January; Feb., February; NA, not applicable.

The patient denied any medical history of gastrointestinal symptoms such as emesis, melena, abdominal pain, or ileus before pregnancy. The patient and her family had no history of previous intestinal cancer. She was screened and was negative for hereditary non-polyposis colorectal cancer syndrome, Crohn’s disease, Peutz-Jeghers syndrome, celiac disease, and cystic fibrosis. Her grandfather had died of hepatic carcinoma.

We prescribed glutathione to improve the patient’s liver function, nifedipine to inhibit uterine contractions, and magnesium sulfate to protect the fetus’s central nervous system. We also administered intramuscular dexamethasone sodium phosphate to promote fetal lung maturation.

During the treatment course, she suffered repeated vomiting that was accompanied by pain in the lower abdomen. Her appetite was thereby affected, and she ate only a little porridge at a time. She emptied her bowels every day, but only in small quantities.

We believed that her vomiting and pain would abate after delivery. She was treated symptomatically with mercoaluminum suspension to protect the gastric mucosa, metoclopramide to settle her stomach, and pethidine hydrochloride to relieve pain as necessary.

A live baby boy weighing 2120 g was delivered prematurely on January 20, 2021. The infant had Apgar scores of 9 in the first minute and 10 in the fifth minute.

**Figure 1:**
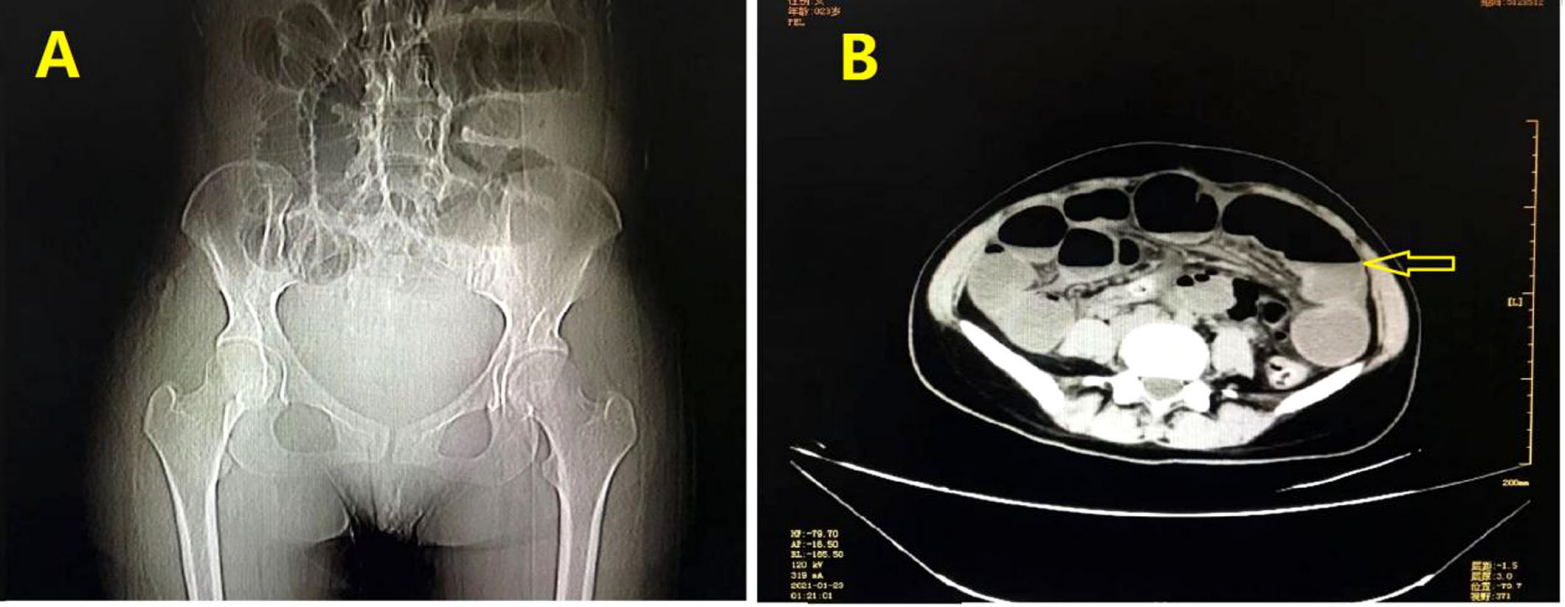
Imaging examination: **(A)** Digital radiology and CT scanning showed gas and fluid accumulation in the small intestine and indicated intestinal obstruction. **(B)** Liquid, air and high intestinal tension were visible (lesion was marked with yellow arrow).

Unfortunately, the patient still had postpartum nausea, vomiting, and abdominal distension and did not defecate for 2 days after delivery. On January 22, all bowel output stopped. Emergency CT indicated a small intestinal obstruction (**Figure 1**). Gastrointestinal decompression and enema treatment were administered.

**Figure 2:**
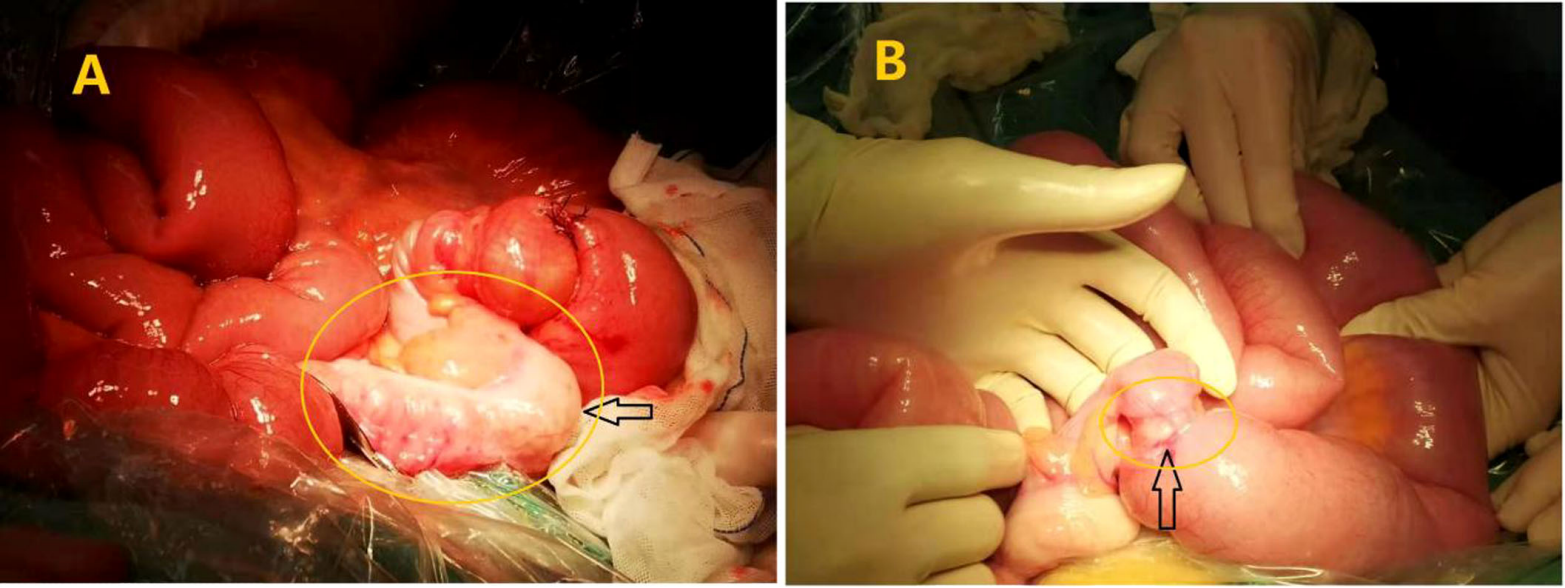
Surgical finding: **(A)** The cancer caused this part of the ileum to become ischemic and pale; **(B)** Annular stenosis in the ileum is indicated by the yellow circles.

There was still no anal discharge after non-surgical management for 2 days. Therefore, exploratory laparotomy with a vertical midline incision had to be performed for obvious abdominal distension. Approximately 200 mL of a yellow effusion was seen in the abdominal cavity. Intraoperatively, there was a hard constricting tumor measuring 2 cm × 2 cm located 5 cm from the ileocecal junction. The intestine was obstructed as evidenced by a dilated proximal bowel with blood, edematous fluid, and intestinal contents and collapsed distal bowel with many enlarged lymph nodes in the mesentery. (**Figure 2**). The specimens were removed from the middle section of the transverse colon about 10cm from the liver curvature of the colon and the terminal ileum about 15cm from the ileocecal valve.

**Figure 3:**
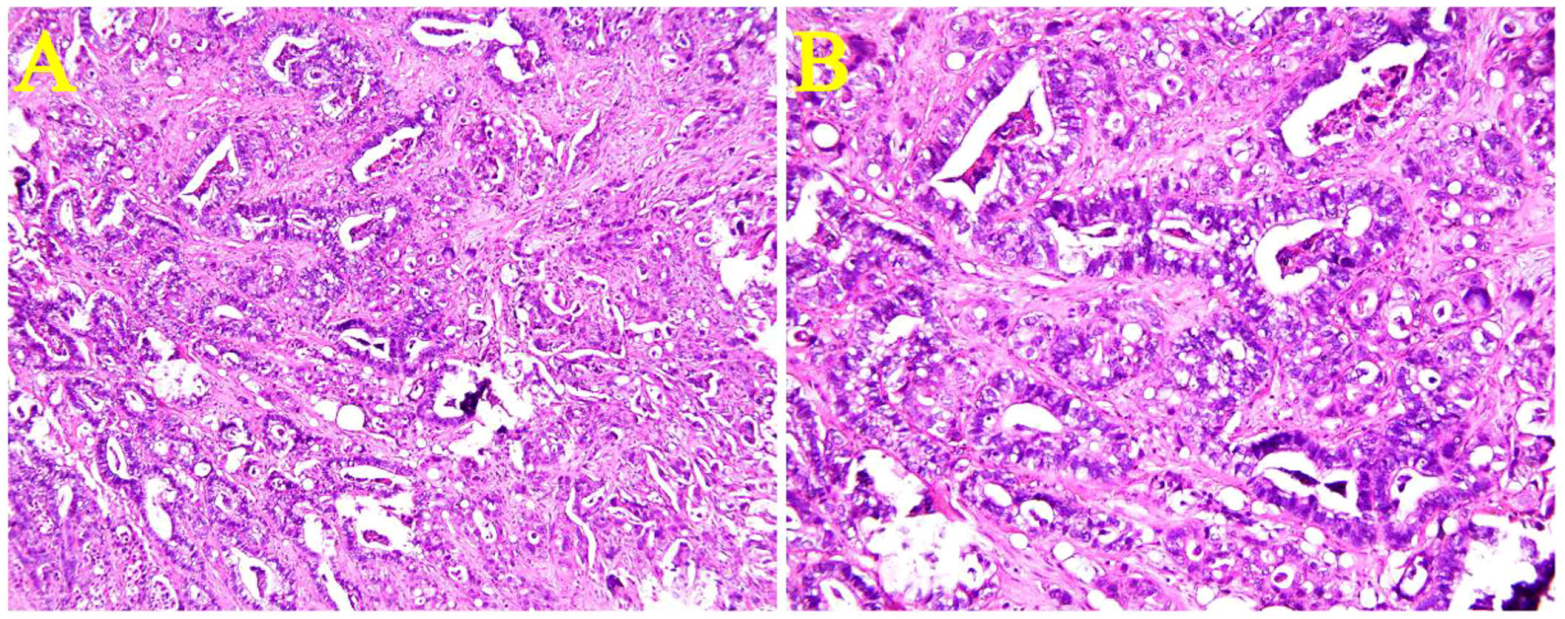
Microscopic view of the ileal adenocarcinoma. An ulcerative high-grade tubular adenocarcinoma had invaded the entire ileal wall. [Hematoxylin & eosin staining, **(A)**×100 and **(B)**×200].

A tumor of approximately 2 cm × 1.5 cm × 1.3 cm in size was removed from the ileum. It was an ulcerative high-grade tubular adenocarcinoma, which had invaded the whole ileal wall (**Figure 3**). Tumor emboli were visible in the blood vessel, but no perineural invasion was observed. Metastases were found in the lymph nodes around the colon and ileum, but no cancer involvement was apparent in the appendix, incisal edge of the ileum, and colon.

The patient was finally diagnosed with adenocarcinoma of the terminal ileum (pT4N2M0), abnormal liver function, gallstones, hypoproteinemia, preterm delivery (35 GWs), and alpha-thalassemia.

**Figure 4:**
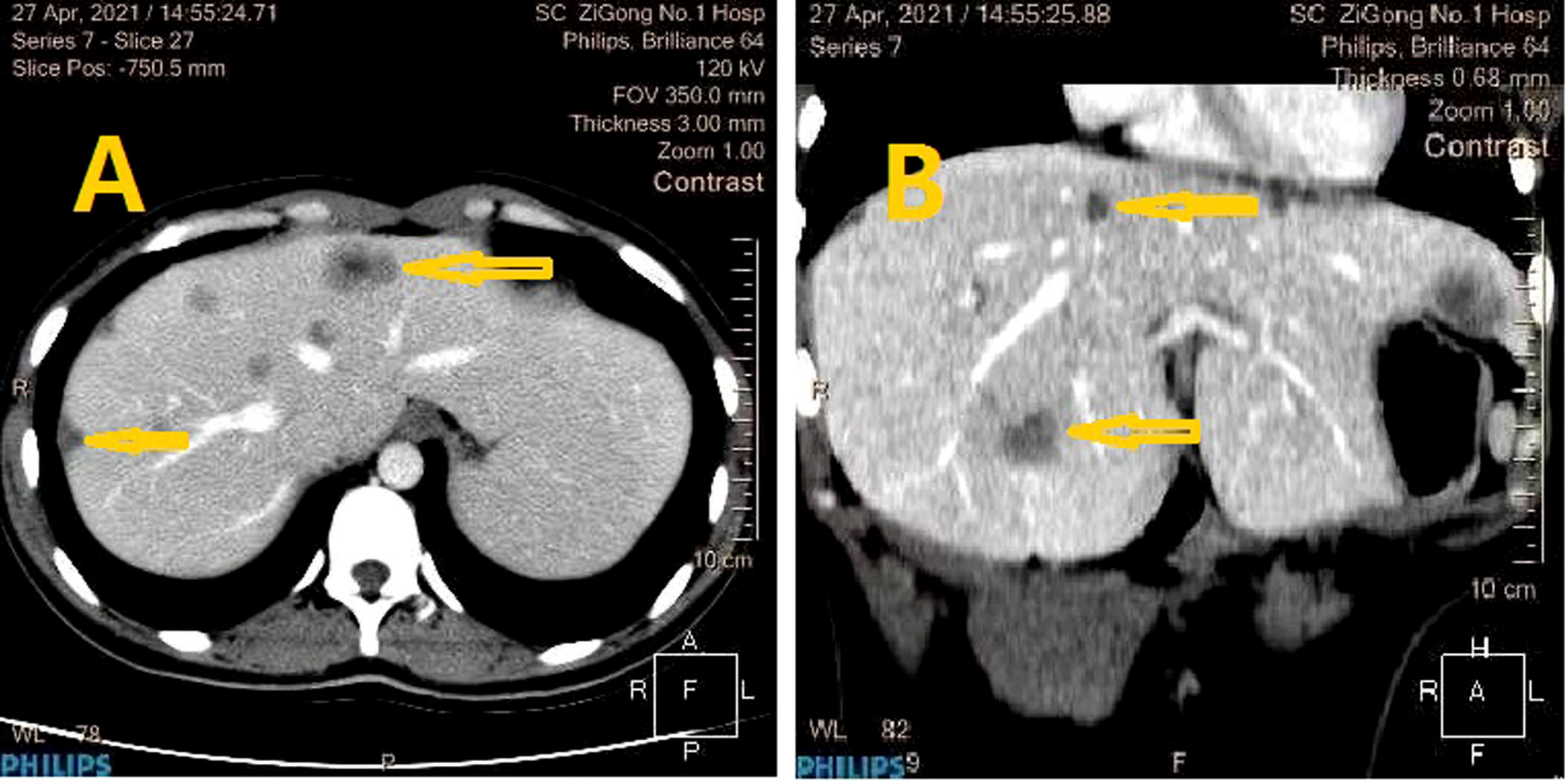
CT imaging: The metastatic liver lesion and low-density lesions in the liver was shown by CT (lesion was marked with yellow arrow) [**(A)**, **(B)** reflect different anatomical layers].

**Figure 5:**
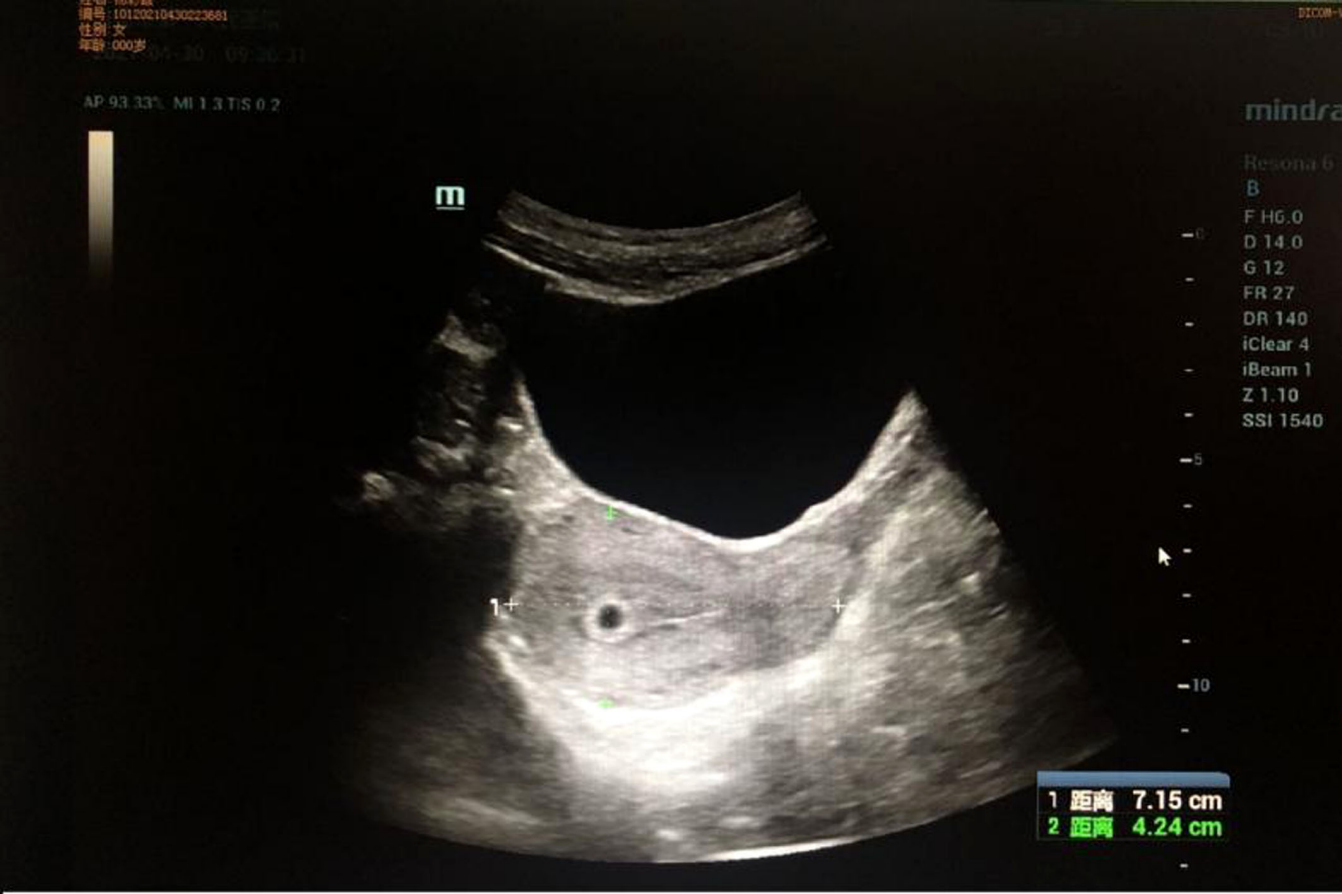
Ultrasound imaging: The gestational sac in the uterus was detected by ultrasound examination.

The patient was discharged on February 3, 2021. Adjuvant chemotherapy was strongly recommended, but she chose traditional Chinese medicine (TCM) instead. She came to our department as an outpatient for a checkup in April 2021. Enhanced CT scanning of her whole abdomen revealed multiple niduses in the liver, the largest being 2.6 cm, which indicated metastatic lesions (**Figure 4**). At the same time, she showed signs of cessation of menstruation, but an intrauterine gestational sac was found by color ultrasonography examination at the last follow-up on April 23 (**Figure 5**). This pregnancy was later spontaneously aborted.

She was hospitalized in the Traditional Chinese Medicine Hospital of Zigong for abdominal pain on July 7, 2021. *Brucea javanica* oil emulsion injection and other TCMs were used against the tumors. She underwent a chemotherapy regimen of oxaliplatin (100 mg) and bevacizumab (260 mg) administered on the first day of the cycle *via* intravenous infusion, plus oral capecitabine, 1 g twice a day for 14 days. This regimen was administered at 3-week intervals. The first, second, third, fourth, fifth, and sixth cycles of chemotherapy began on July 17, August 7, September 6, September 29, October 20, and November 15, 2021, respectively. CT revealed that the metastases in the liver were necrotic, but the metastases in the lymph nodes of the abdominal cavity had not changed. The patient’s last treatment was pelvic tumor angiography and perfusion chemotherapy with irinotecan.

She suffered constant abdominal pain, and her weight dropped to 35 kg because her appetite was poor. She died on February 26, 2022.

## Discussion

Small bowel adenocarcinomas account for only 5% of all gastrointestinal malignancies (5). A retrospective analysis showed that the incidence of small intestinal tumors was 60.6% in the duodenum, 20.7% in the jejunum, and 18.7% in the ileum. The median age of patients was 63 years (range, 23–90 years) (6), which is consistent with the findings of Colina et al. (7).

The rising incidence of IA has been linked to age and alcohol consumption, a high-fat diet, high-sugar diet, smoked foods, and red meat. In contrast, a high intake of fish, coffee, fruits, and vegetables may reduce the risk (8–10). In most studies, the risk factors for IA are Lynch syndrome, inflammatory bowel disease, Crohn’s disease, and familial adenomatous polyposis (6, 8). Our case had a negative history for these risk factors.

The most common clinical manifestations of IA are weight loss, vomiting, cramping abdominal pain, and occult gastrointestinal bleeding, but perforation is rare (8, 10–13). Vomiting and nausea may persist throughout a pregnancy in approximately 10% of affected women (14), so these symptoms are easily attributed to pregnancy. Our patient presented with vomiting (a typical symptom of pregnancy) and pain, which represented a threat of premature abortion. For pregnant women, inadequate weight gain is similar to weight loss in the general population.

Flexible enteroscopy performed with balloon-assisted or spiral techniques to explore the terminal ileum is a predominant and effective method for diagnosing IA, but it is less accurate than CT enteroclysis or video capsule endoscopy (VCE) (6, 10, 15). However, CT and contrast agents are contraindicated in pregnant women. VCE has the advantages of being simple, safe, reliable, and of short duration, and requires no anesthesia. Endoscopic indications during pregnancy are severe or refractory nausea and vomiting or abdominal pain according to the American Society for Gastrointestinal Endoscopy (16). The primary contraindications to VCE are known or suspected intestinal obstruction, strictures, fistulas, cardiac pacemakers, and swallowing disorders. Pregnancy is a relative contraindication according to the US Food and Drug Administration (16). Thus, pregnancy represents a dilemma for the diagnosis of IA. Under the surveillance of a multidisciplinary team, endoscopic examination could be performed safely. The advantage of magnetic resonance imaging (MRI) is that it does not expose the patient to radiation, and its use in this setting has been reported (17–19). To date, unfortunately, almost all tumors in the jejunum and ileum have been detected during emergency surgery because of obstruction, perforation, or gastrointestinal bleeding, as in our patient. In retrospect, the indications in our case were sufficient for endoscopy or MRI evaluation. At least the upper digestive tract lesions can be ruled out by gastroscopy. After the upper digestive tract lesions are excluded, MRI can be considered to further explore the presence of lesions in the lower digestive tract. Such a procedure would keep safe for both the pregnant woman and the fetus, while also finding hidden lesions as much as possible.

Segmentectomy of the ileum and colon is the only cure for IA. Because lymph node metastasis strongly affects the prognosis, at least eight regional lymph nodes must be retrieved intraoperatively for evaluation (20). The combination of leucovorin, 5-fluorouracil, and oxaliplatin (FOLFOX) is the regimen most frequently used (6, 8). Cetuximab or panitumumab should not be used to treat small bowel adenocarcinoma (SBA) because it is clinically useless in RAS wild-type cases (6, 8). After systemic treatment, the patient should be closely monitored through physical examination and measurement of carcinoembryonic antigen or carbohydrate antigen 19-9 levels, or both. CT scanning of the chest, abdomen, and pelvis is necessary.

When malignancy is diagnosed during pregnancy, it is generally found at advanced stages (21). The lack of screening programs enhances the difficulty of detecting IA in particular. Huffman et al. reported that advanced age, advanced stage, and a lymphocyte-to-monocyte ratio of <1.56 were independent predictors of survival in cases of resectable SBA (22). The rate of recurrence of IA is as high as 77%, far higher than that of duodenal (54%) and jejunal adenocarcinomas (65%) (7). The prognosis is poor because IA is usually found at an advanced stage.

In our patient, IA caused only incomplete ileus, which was relieved after symptomatic treatment until the typical symptoms of ileus appeared after delivery. When the initial treatment of ileus failed, emergency surgery was needed, and only then was IA discovered unexpectedly. This is consistent with the report by Dabaja et al. that all cases of IA require emergency management (4).

We propose an “IA triad” definition of refractory vomiting, vague abdominal pain, and weight loss (or inadequate weight gain in pregnant women). We believe that this IA triad can alert medical professionals to the possibility of a malignant tumor in young pregnant patients. Timely detection of tumors may contribute to earlier staging, which strongly affects the prognosis. To avoid misdiagnosis in similar cases, attention should be paid to important symptoms, including refractory vomiting, vague abdominal pain, and weight loss (or inadequate weight gain in pregnant women). Perhaps in the near future, artificial intelligence modules in medical management systems can help us solve these problems (23).

## Conclusion

Pregnant women who experience symptoms of intestinal obstruction should be alert to the possibility of small intestinal malignancy. IA is an insidious tumor in pregnant women. An IA triad can be defined as refractory vomiting, vague abdominal pain, and weight loss (or inadequate weight gain in pregnant women). Pregnant women with the IA triad should undergo investigation with endoscopy or, if necessary, magnetic resonance imaging.

## Data availability statement

The original contributions presented in the study are included in the article/supplementary material. Further inquiries can be directed to the corresponding authors.

## Ethics statement

Ethical review and approval was not required for the study on human participants in accordance with the local legislation and institutional requirements. The patients/participants provided their written informed consent to participate in this study. Written informed consent was obtained for the publication of this case report.

## Author contributions

CX was responsible for the conceptualization, data collection and original manuscript drafting. QC and CC reviewed the literature. CCand XX corrected the data and revised the manuscript. YZ collected pathological data and revised the manuscript. All authors have read and approved the final manuscript.
